# Influence of Insolation on the Efficiency of NO_3_ Removal from Wastewater Treated in the Hydroponic System

**DOI:** 10.1007/s11270-018-3888-9

**Published:** 2018-06-29

**Authors:** Aleksandra Bawiec, Katarzyna Pawęska, Krzysztof Pulikowski, Joanna Kajewska-Szkudlarek

**Affiliations:** Institute of Environmental Engineering, Wrocław University of Environmental and Life Sciences, 24 Grunwaldzki Sq., 50-363 Wrocław, Poland

**Keywords:** Nutrients removal, Wastewater treatment, Hydroponic lagoon, Efficiency of wastewater treatment, Municipal wastewater

## Abstract

The use of plants and natural processes for wastewater treatment is an issue that arouses interest among technologists and scientists around the world. The aim of the article was to analyze the influence of the air temperature and insolation on the removal of nitrate nitrogen from the wastewater treated in the hydroponic system, under greenhouse conditions. Samples of sewage for its quality tests were taken from a wastewater treatment plant (WWTP) located in the southwestern part of Poland. Data regarding daily sunshine duration and average daily air temperature values in selected periods of 2013–2016 come from a meteorological station located 30 km from WWTP. The conducted research and analyses of the results clearly indicate that under moderate climate conditions, the amount of solar radiation reaching the Earth’s surface is insufficient to ensure the year-round, effective wastewater treatment process in the hydroponic system. In the case of air temperature, no correlation was found between the tested parameters, which indicates the lack of temperature influence on the efficiency of NO_3_ removal from the wastewater by macrophytes growing in the lagoon.

## Introduction

The growing problem of eutrophication, affecting not only lakes and dam reservoirs, but also coastal regions of the oceans and seas, is largely caused by the discharge to the receiving water bodies of insufficiently treated wastewater that comes from various sources (Babin et al. 2015; Dąbrowska et al. 2017; Liu et al. 2018). Even after wastewater treatment in the traditional systems, there is a risk of remaining in the sewage the traces of toxic chemicals and nutrients (Zhao et al. 2015; Gizińska-Górna et al. 2016). Not without significance is also the increasing load of discharged pollutants, which results from the constantly increasing amount of discharged sewage. This is related to the intensive development of industry and the expansion of residential areas, which is closely related to the continuous growth of population (Zhi et al. 2016; dos Santos et al. 2018).

The constant search for effective methods of removing excess nutrients from sewage has led to the development of various technologies using naturally occurring purification processes. The most popular of them are constructed wetlands (with surface and sub-surface vertical or horizontal flow), wastewater stabilization ponds, for example *Lemna minor* systems, as well as different kinds of hydroponic systems (Saggaï et al. 2017; Sarkar et al. 2017; Gatidou et al. 2017; Bawiec 2018).

Hydroponic systems are based on the creation of environmental conditions similar to boggy ecosystems, or even river ecosystems, in which both animal and plant aquatic organisms play a major role in the treatment processes. In the case of watercourses, which are often the models for the hydroponic systems, it is based on the occurrence of water self-purification. This process depends on the morphological characteristics of the watercourse and is based on the interaction of physical, chemical, biological, and hydraulic factors striving to maintain the balance of the aquatic ecosystem (Das et al. 2016).

Biological factors affecting the effectiveness of the process of self-purification of water include the activities of organisms that inhabit the water ecosystem—mainly bacteria, as well as algae, macrophytes, and animal organisms. Bacteria carry out the processes of organic matter decomposition to simple mineral compounds, which can be the substrates for life cycles of producers such as algae and macrophytes (Simcčič and Germ 2009). In addition to bacteria that conduct in the aquatic environment also the processes of nitrogen transformation through nitrification and denitrification, the ability of plants to collect and accumulate nitrogen and phosphorus began to be used. Macrophytes (emergent, submergent, floating) also play the role of producers that are of great importance in the environment for the biotic structure and the functioning of aquatic ecosystems (Hu et al. 2014). As producers, they absorb the carbon dioxide and with the participation of solar energy, they produce oxygen as a result of photosynthetic transformation (Graham et al. 2009). In addition, their roots immersed in the water provide an excellent environment for the existence of fish and the growth of bacteria that conduct purification processes (Hu et al. 2014).

The most important factors affecting the condition of plants growing in the hydroponic systems are the availability of nutrients in the aqueous solution and meteorological conditions, including temperature and light regimes (Shaban et al. 2016). Solar radiation is the basic factor that determines the growth and proper development of plants by affecting the synthesis of many key substances (Wojciechowska et al. 2015). The photosynthetic, photomorphogenetic, and phototropic answer depends, among others on the quality and quantity of radiation reaching the Earth’s surface and the photoperiod (Kurilčik et al. 2008). Proper lighting conditions determine the effective use of assimilated CO_2_ for the production of biomass and regulation of the plant’s water balance (Brazaityte et al. 2006). The occurrence of the light stress or shadows may lead to changes in the course of basic physiological processes (Hartman et al. 2014). The spectrum of the radiation reaching the surface of leaves is also of great importance for the condition of plants. In the beam of the solar radiation reaching the Earth’s surface, the emission of the blue, red, and green light can be distinguished as well as the remaining bands of visible and invisible light, differing in their wavelength. The most important for photosynthesis are the blue and red beams of the emitted waves, and together with the other components of the visible beam of radiation affect the shape of plants, their growth, and photomorphogenesis (Singh et al. 2015).

The sunshine duration that means the time of direct solar radiation inflow to the Earth’s surface, is, apart from the air temperature and precipitation, one of the basic parameters measured by meteorological stations and used in research on various topics. PAR—photosynthetically active radiation with a wavelength in the range of 400–700 nm, directly affects the growth and condition of plant organisms (Fletcher et al. 2013; Kuwahara and Taguchi 2015). The total solar radiation reaching the Earth’s surface consists of direct and diffuse radiation, which, when falling on the water surface, is subject to the additional reflection (Lampert and Sommer 1996). In the case of aquatic ecosystems, the availability of light contributes to the increase in temperature in the photic zone, which intensifies the process of self-purification of water by accelerating the life processes of organisms responsible for the mineralization of organic matter (Jarosiewicz 2007).

The paper attempts to assess the effect of insolation on the efficiency of nutrients removal from wastewater treated in the hydroponic system used as the third stage of wastewater treatment in order to excess nitrogen and phosphorus removal.

## Materials and Methods

### Object Description

The research object is the Municipal Wastewater Treatment Plant (WWTP) located in the municipality in the south-western part of Poland, 50° 27′ N, 17° 00′ E. The size of the WWTP is 22,396 p.e., and the design capacity is 2500 m^3^ d^−1^. The daily sewage inflow is smaller than planned and is around 1350 m^3^ d^−1^. About 500–700 m^3^ are wastewater delivered from cesspools combined with the sludge from septic tanks. The receiver of purified wastewater is the Nysa Kłodzka River.

**Fig. 1:**
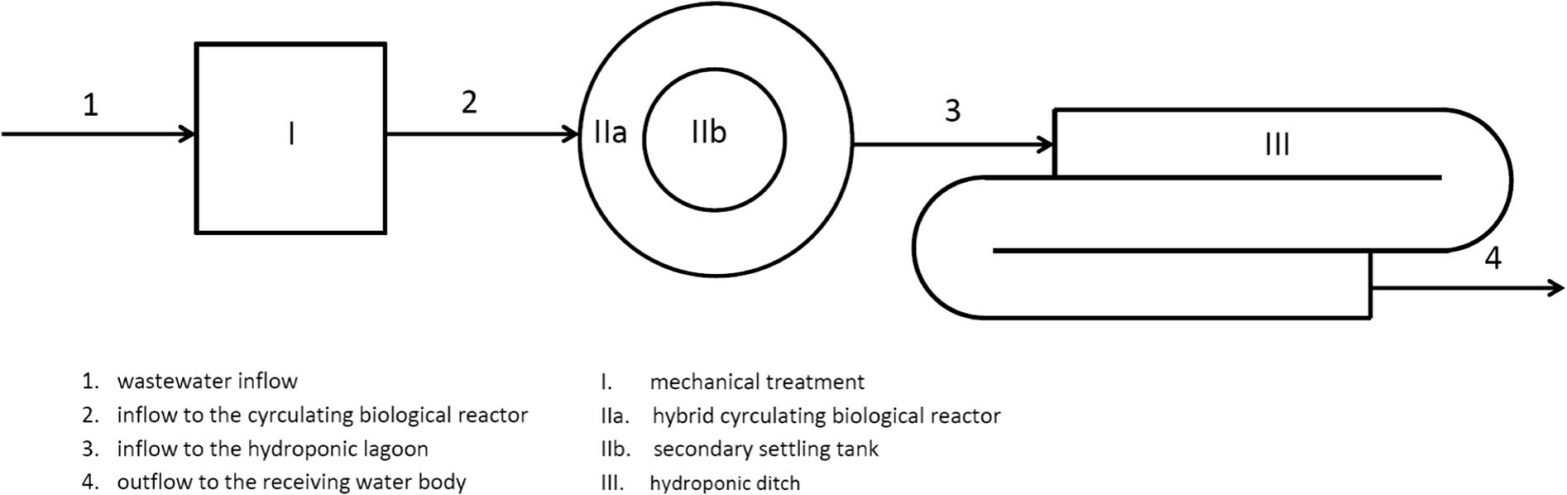
Block diagram of the wastewater treatment processes in the WWTP

This object is built on a circular plan, and the whole process of the wastewater treatment is closed under a roof. The treatment process consists of three individual stages—mechanical and biological treatment as well as the additional purification in the hydroponic lagoon. The block diagram of the wastewater treatment process is shown in Fig. 1.

The mechanical treatment (I) consists of screens and a separator. Biological treatment takes place in a hybrid circulating biological reactor (IIa), using both methods—the activated sludge and the biological membrane. It is possible thanks to the use of vertical panels immersed in the wastewater, separating oxygen, hypoxic and anaerobic zones of the reactor from each other. After sedimentation of excess sludge in the secondary settling tank (IIb), clarified sewage is directed to the hydroponic lagoon, which is the third stage of the wastewater treatment (III).

**Fig. 2:**
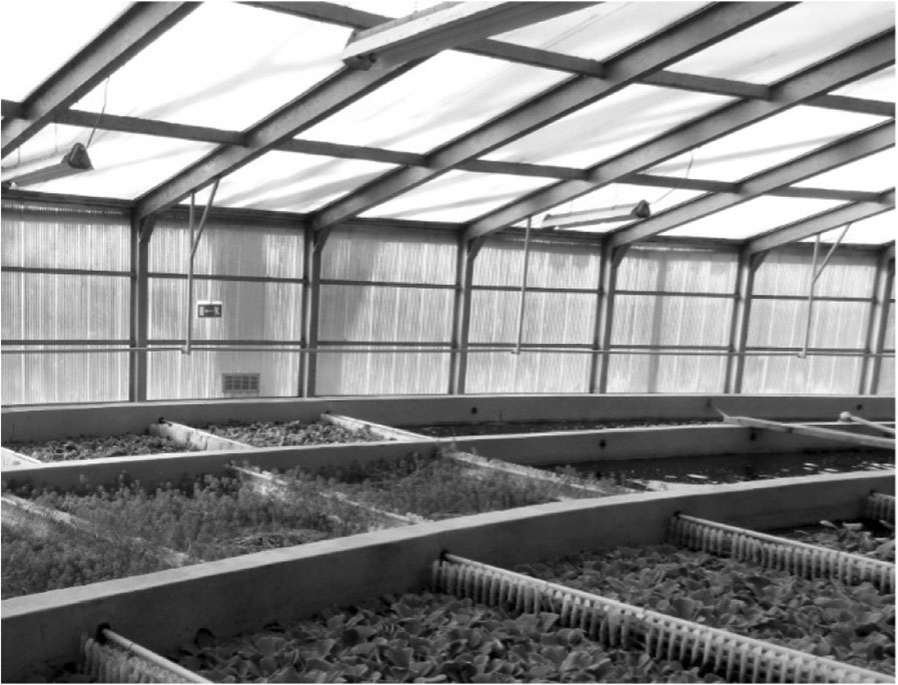
Hydroponic lagoon located under the roof made of polycarbonate sheets [fot. A. Bawiec]

The hydroponic lagoon is a concrete ditch with a semi-circular bottom, whose total length is 191 m, width 2.1 m, and the average depth—1.4 m. The designed time of the sewage flow through the lagoon is about 9 h. The ditch is built in the shape of an artificial river, in which partial aeration of sewage is applied. On the surface of the wastewater there are plastic floating panels that provide support for the planted macrophytes, i.e., *Pistia tratiotes*, *Myriophyllum verticillatum* and in the vast minority *Eichhornia crassipes* and *Limnobium laevigatum.* The task of planted vegetation is the uptake of nitrogen and phosphorus compounds before discharge of sewage to the river, as well as the creation of appropriate conditions for the growth of bacteria and other aquatic organisms involved in self-purification processes. In order to provide the best conditions for plants life processes, including photosynthesis, the sewage purification in the lagoon is conducted under greenhouse conditions. The walls and the roof of the room where the hydroponic bed is located are made of polycarbonate sheets. Polycarbonate allows penetration of the solar radiation, which provides the light access for plants and the ambient air heating, and is also a material with high thermal insulation, resistant to weather conditions (Janjai et al. 2011). A photo of the hydroponic ditch with polycarbonate cover is shown in Fig. 2.

In the case of the receiver of treated sewage which is the Nyska Kłodzka River, the effective removal of biogenic compounds—nitrogen and phosphorus—from the wastewater is of particular importance. This is due to the fact that on the river are located retention reservoirs, whose waters are used for energy and recreational purposes. Ensuring high quality of water in reservoirs with limited susceptibility to eutrophication is of key importance for the possibility of its later use (Wiatkowski 2015).

### Sample Collection

Samples of sewage were collected once a month from two points of the hydroponic lagoon—at the inlet to the lagoon (biologically purified sewage) and from the outlet of the lagoon (outflow to the receiver). Samples of 1.5 dm^3^ volume were transported in polyethylene terephthalate (PET) containers to the laboratory of the Faculty of Environmental Engineering and Geodesy, Wroclaw University of Environmental and Life Sciences. The transport time of unfixed samples was less than 3 h. In each of the samples, the content of nutrients, i.e., nitrates and phosphate concentrations were measured according to the methods and standards set out in the Table 1.

**Table 1:** List of standards and methodology of tested indicators

No	Pollutant indicator	Methodology of research	Standard
1	Nitrates	Spectrophotometric method	PN-82C-04576/08
2	Phosphates	Spectrophotometric method	ISO 6878/1:2006

The basic data regarding the daily sums of sunshine duration and average daily values of air temperature in selected periods of 2013–2016, which were used for analyses, came from a meteorological station located in Kłodzko (approx. 30 km from the WWTP). The station is a part of the measurement network of the Institute of Meteorology and Water Management – National Research Institute (IMGW-PIB).

The effectiveness of mineral form of nitrogen removal in the hydrobotanic treatment plants is closely related to meteorological conditions, in particular the amount of light reaching the plants and the temperature of the air (Shaban et al. 2016). Therefore, the influence of these factors on the obtained reduction of nitrate concentration was analyzed. Values of these parameters were analyzed in the period preceding the day where the composition of sewage was determined. The sum of insolation for periods from 1 to 30 days preceding the day of the analysis was determined. In the case of insolation, this dependence is as follows:

$$ {U}_n=f\left(\sum \limits_{i=n-k}^{i=n-1}U\right)\ \left[\%\right] $$

where

*U*_*n*_: sum of insolation in the period preceding the day of determining the composition of sewage, *h*;
*U*: daily sum of insolation, *h*;
*k*: the length of the period for which the sum insolation was taken into account (1, 30).

Then a statistical analysis of the relationship between the effectiveness of nitrates removal from the wastewater and the sum of insolation in the period preceding the analysis of wastewater composition was carried out. The calculations were carried out for periods from 1 to 30 days.

Statistical analyses were performed using software STATISTICA.

## Results

The results of nitrate concentration measurements in the samples collected from the inlet to the lagoon and the outlet from the lagoon in each year of period of research are presented in the Table 2.

**Table 2:** Concentrations of the N-NO_3_ in the wastewater from the inflow and the outflow of the hydroponic lagoon

	Inlet to the lagoon	Outlet of the lagoon
	NO_3_ [mg N-NO_3_ dm^−3^]	
2013		
Average	1.15	1.14
Min.	0.03	0.06
Max.	3.54	3.81
Stand. dev.	1.42	1.43
2014		
Average	1.09	1.09
Min.	0.06	0.10
Max.	2.53	2.72
Stand. dev.	0.92	0.92
2015		
Average	2.64	2.17
Min.	0.37	0.12
Max.	6.16	4.44
Stand. dev.	1.56	1.33
2016		
Average	3.51	3.27
Min.	2.14	1.94
Max.	5.42	4.92
Stand. dev.	0.91	0.81

**Fig. 3:**
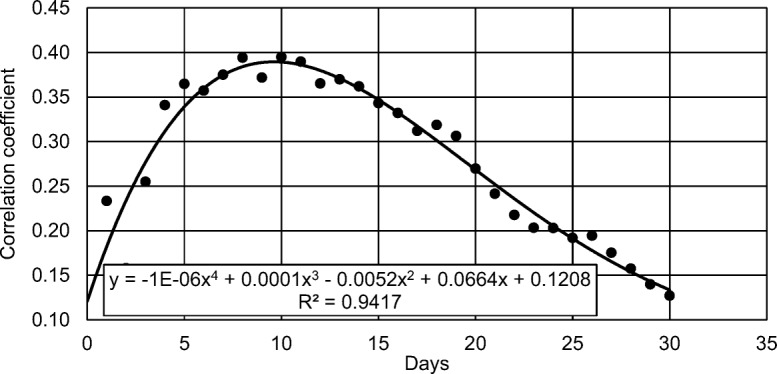
Correlation coefficient between the efficiency of nitrate nitrogen removal and the length of the period preceding the analysis day for which the sum of insolation was calculated

The results of statistical analyses of the correlation between the removal of nitrates and the sum of insolation in the period preceding the analysis are shown in Fig. 3. Substantial relationships (for *p* = 0.05) were obtained only for the length of periods of 8, 10 and 11 days. Based on the trend line, it was assumed that the greatest effect on the effectiveness of nitrate removal in the hydroponic treatment plant has the value of insolation in the 10-day period before making the analyses.

A similar procedure was conducted for the air temperature, but in this case no statistically significant dependencies were obtained. The obtained values of the correlation coefficient ranged from − 0.03 to − 0.16. Noteworthy is the fact that in all 30 cases, negative correlation coefficients were obtained, which may indicate that the temperature increase is more conducive to the intensification of the nitrification process than to the uptake of this form of nitrogen by plants planted in the hydroponic lagoon.

**Fig. 4:**
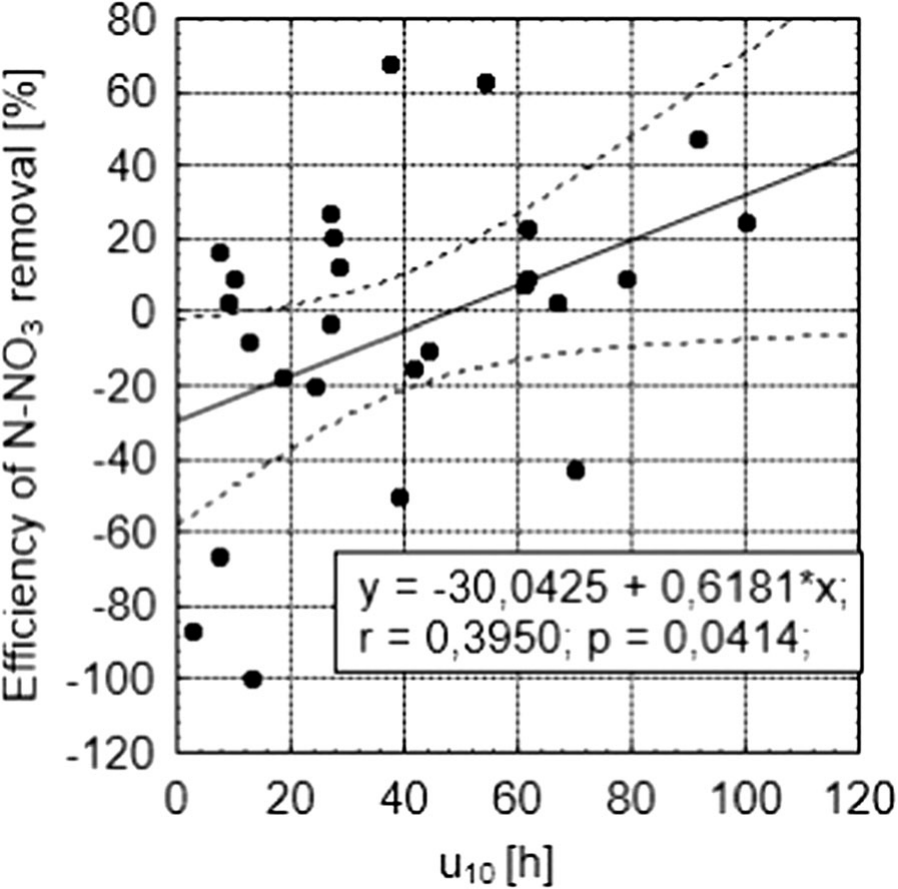
The dependence of NO_3_ removal efficiency on total sunshine duration from the previous 10 days

Figure 4 shows the relationship between the effectiveness of nitrate removal and the sum of insolation during the 10 days preceding the analysis. The obtained dependence is significant for *p* = 0.04, and the correlation coefficient is 0.40. It is worth noting that the value of nitrogen removal efficiency for low values of insolation sum within 10 days takes negative values, so the amount of radiation reaching plants is insufficient. On the basis of this graph, the minimum value of the 10-day sum of insolation was determined. The sum of insolation that is necessary to obtain the effect of nitrates removal from the wastewater treated in the hydroponic lagoon is 48.6 h (Fig. 4).

A similar calculation was carri ed out to analyze the effect of insolation on the efficiency of phosphate removal in the hydroponic system. The low values obtained for the correlation coefficient *k* indicate that there is no dependence between the sum of insolation and PO_4_ concentration in the wastewater.

**Fig. 5:**
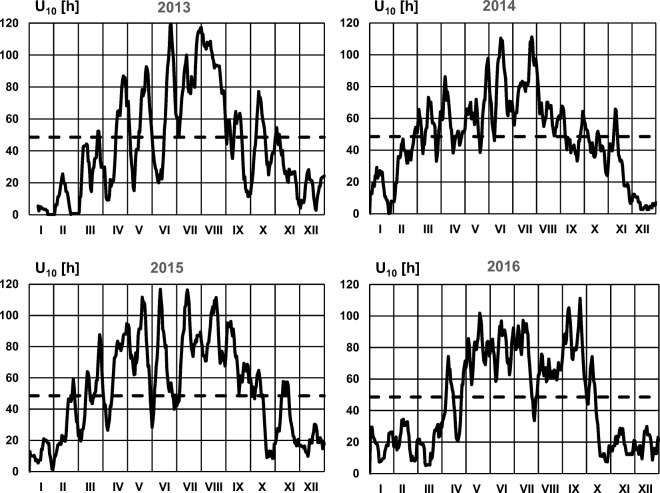
Days in 2013–2016, for which the sum of insolation in 10 days preceding them is higher than 48.6 h

**Fig. 6:**
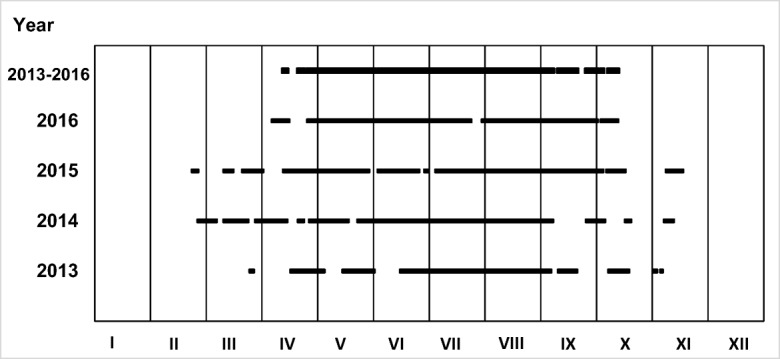
Periods in which in 3 out of 4 analyzed years the sum of insolation from 10 days exceeding 48.6 h was obtained

For the years 2013–2016, days in which the sum of insolation for the decade preceding the analyses of the sewage composition is higher to 48.6 h were determined (Fig. 5), and thus periods in which it is possible to remove nitrates from the wastewater in the hydroponic lagoon (Fig. 6). In order to determine universal values for the entire period 2013–2016, it was assumed that the value is achieved for the whole period, if it was achieved for 3 out of 4 analyzed years. The results of calculations are presented in Fig. 6.

## Discussion

The availability of solar radiation for plants inhabiting the hydroponic lagoon is a key factor enabling their development and, consequently, the uptake of nitrate nitrogen from the wastewater. Hydroponic systems are most commonly used for the production of arable crops. Research on the optimal conditions for the growth of crops cultivated in hydroponic conditions has shown that the solar radiation is necessary to obtain maximum yields of high-quality plants, but it is problematic to determine the optimal level of insolation (Choi et al. 2014). Shading of cultivated plants and reducing the intensity of light reduces the photosynthetic assimilation, and in extreme cases can lead to damage to the chlorophyll contained in the leaves (Critchley and Smillie 1981). Exposing plants to intense sunlight may cause serious damage to the leaves (chlorosis, necrosis, leaf twisting) (Suzuki et al. 2014). Autotrophs require, in addition to light, the right amount of nitrogen in an absorbable form to effectively uptake CO_2_ necessary for photosynthesis and phosphorus via cross-membrane transport and also to maintain the enzymatic activity (Borer et al. 2013). Thus, insufficient lighting of the surface of the hydroponic lagoon, may not only affect the deterioration of the condition of the plants, but may also be the reason for the limited ability to uptake the nitrogen and phosphorus from the wastewater.

## Conclusions

The conducted research and analysis of the results clearly indicate that under moderate climate conditions, the amount of solar radiation reaching the Earth’s surface is insufficient for year-round, effective treatment of wastewater in the hydroponic systems. In the periods from January to April and from October to the end of December, it is necessary to provide additional light from artificial sources. However, this causes an increase in financial expenses incurred on wastewater treatment, and thus forces the search for energy-saving sources of light and the possibility of obtaining energy for their supply from natural renewable sources.

As a result of the analysis of the research, there were no statistically significant relationships between the air temperature and the efficiency of NO_3_ removal from wastewater, which may indicate that the temperature increase is more conducive to the intensification of the nitrification process than the absorption of nitrogen by macrophytes.

In the case of the wastewater treatment processes using plants, ensuring adequate lighting is a key issue. However, attention should be paid to the fact that in the hydroponic lagoon, due to intensive wastewater lighting, intensive growth of algae may occur. Algae may additionally contribute to the reduction of nitrogen and phosphorus loads; however, in systems with the hydroponic lagoon, they may increase the concentration of the total suspended solids in the outflow. Therefore, it is of great importance to search not only for energy-saving methods of wastewater lighting, but also for optimal time of exposure. This approach to the problem requires further verification and is the direction of further research carried out by the authors of this article.
